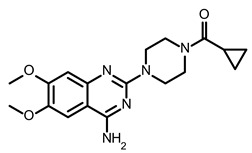# Structural and Functional RNA Motifs of SARS-CoV-2 and Influenza A Virus as a Target of Viral Inhibitors

**DOI:** 10.3390/ijms24021232

**Published:** 2023-01-08

**Authors:** Izabela Szczesniak, Agnieszka Baliga-Gil, Aleksandra Jarmolowicz, Marta Soszynska-Jozwiak, Elzbieta Kierzek

**Affiliations:** Institute of Bioorganic Chemistry, Polish Academy of Sciences, Noskowskiego 12/14, 61-704 Poznan, Poland

**Keywords:** SARS-CoV-2, influenza A virus, COVID-19, RNA structure, antiviral strategies, RNA interference, antisense oligonucleotides, small molecules

## Abstract

Severe acute respiratory syndrome coronavirus 2 (SARS-CoV-2) is responsible for the COVID-19 pandemic, whereas the influenza A virus (IAV) causes seasonal epidemics and occasional pandemics. Both viruses lead to widespread infection and death. SARS-CoV-2 and the influenza virus are RNA viruses. The SARS-CoV-2 genome is an approximately 30 kb, positive sense, 5′ capped single-stranded RNA molecule. The influenza A virus genome possesses eight single-stranded negative-sense segments. The RNA secondary structure in the untranslated and coding regions is crucial in the viral replication cycle. The secondary structure within the RNA of SARS-CoV-2 and the influenza virus has been intensively studied. Because the whole of the SARS-CoV-2 and influenza virus replication cycles are dependent on RNA with no DNA intermediate, the RNA is a natural and promising target for the development of inhibitors. There are a lot of RNA-targeting strategies for regulating pathogenic RNA, such as small interfering RNA for RNA interference, antisense oligonucleotides, catalytic nucleic acids, and small molecules. In this review, we summarized the knowledge about the inhibition of SARS-CoV-2 and influenza A virus propagation by targeting their RNA secondary structure.

## 1. Introduction

Severe acute respiratory syndrome coronavirus 2 (SARS-CoV-2) causes coronavirus disease 2019 (COVID-19). On 11 March 2020, the World Health Organization (WHO) declared COVID-19 a global pandemic that was responsible for a lot of infections and deaths all over the world. Moreover, plenty of people struggled with disruptions to health services, travel, trade, and education; in addition, COVID-19 had a negative impact on people’s physical and mental health [1,2]. The most recent global pandemic before COVID-19 was the 2009 influenza A (H1N1) pandemic (formerly known as swine flu). The 2009 H1N1 virus possessed a unique combination of segments from human, swine, and avian influenza A viruses and swept the globe with rapid speed [3]. In the 20th century, three such influenza pandemics occurred. The most devastating was the Spanish flu (H1N1strain) in 1918, which caused the deaths of over 50 million people [4,5]. In 1957, “Asian flu” was responsible for more than 1 million deaths [6]. Next, the H3N2 “Hong Kong flu” in 1968 resulted in 0.75–1 million deaths [7,8]. It is an escalating threat that a new pandemic will occur [9].

The SARS-CoV-2 and influenza viruses are RNA viruses [10,11]. SARS-CoV-2 is an approximately 30 kb, positive sense, 5′ capped single-stranded RNA virus. Moreover, subgenomic RNAs are produced during discontinuous transcription, a process which is characteristic of the Coronoviridae family [12,13]. The influenza A virus possesses eight single-stranded negative sense segments [14].

The RNA secondary structure in the untranslated and coding regions is crucial in the viral replication cycle [15]. It is credited with a role in RNA–RNA interactions, protein binding, and the recruitment of modifying enzymes that evade host defense mechanisms [16]. The virus RNA structure controls processes such as splicing; switching between transcription and replication and RNA packaging are controlled by the virus RNA structure [17]. The secondary structure within the RNA of the SARS-CoV-2 and the influenza viruses has been intensively investigated. The bioinformatic study revealed that the SARS-CoV-2 genome has almost twice the tendency to create a secondary structure than the HCV genome, one of the most structured RNA genomes in nature [18,19]. The bioinformatic prediction of the secondary structure models for the extended 5′ untranslated region (5′UTR), the frameshifting stimulation element, the 3′ untranslated region (3′UTR), and the regions of the SARS-CoV-2 viral genome that have a high propensity for RNA secondary structures and are conserved within SARS-CoV-2 strains were published [19,20]. Subsequent bioinformatics analyses showed that the regions creating the highest amount of structure within the SARS-CoV-2 genome are in the 5′ end and the regions corresponding to glycoproteins S and M [21]. Recently, RNA structure mapping of the complete SARS-CoV-2 genome and subgenomic RNA in vitro, in vivo, and in cellulo were published [22,23,24,25,26,27,28]. Moreover, the 3D folding of selected domains and motifs of genomic RNA was also proposed [29].

For the influenza A virus (IAV), there are predicted RNA structural motifs [17,30,31,32,33,34,35]. Some structural RNA motifs were experimentally determined in vitro [34,36,37], and a compensatory mutagenesis study of the RNA structural motifs of the influenza A virus was published [38,39,40]. Recently, in vitro secondary structure models based on experimental data were proposed for full-length vRNA5, vRNA7, and vRNA8 of A/Vietnam/1203/2004 (H5N1) and vRNA5 and vRNA8 of A/California/04/2009 (H1N1) [41,42,43,44,45], as well as segment 5 (+) RNA of A/Vietnam/1203/2004 [46]. A secondary structure for vRNA8 of A/California/04/2009 (H1N1) formed in the presence of cellular and viral components was proposed [47]. To date, the in vivo analysis of influenza A mRNA secondary structures, the in virio selective 2′-hydroxyl acylation analyzed by primer extension and mutational profiling (SHAPE-MaP) of the whole influenza genome of A/WSN/1933 (H1N1), and the in virio studies on the structures and interactions of A/Puerto Rico/8/34 (H1N1) and A/WSN/1933(H1N1) have been published [48,49].

Both the SARS-CoV-2 and the influenza A viruses attack the respiratory tract, cause similar symptoms, and provoke epidemics and pandemics, and it happens that the infections occur simultaneously [50]. Furthermore, Bai showed that coinfection with the influenza A virus enhances SARS-CoV-2 infectivity in a broad range of cell types [51]. For this reason, the research and public interest often focus on both of these RNA viruses. This review responds to this interest. Most of the designed inhibitors target viral proteins [52,53]. However, because the whole of the SARS-CoV-2 and influenza virus replication cycles are dependent on RNA with no DNA intermediate, RNA is a natural and promising target for the development of inhibitors. The knowledge about the secondary structure of the RNA of the SARS-CoV-2 and the influenza viruses could be used to inhibit the propagation of the viruses. There are a lot of RNA-targeting strategies for the regulation of pathogenic RNA, such as small interfering RNA (siRNA) for RNA interference, antisense oligonucleotides (ASO), catalytic nucleic acids, and small molecules (SM). In this review, we summarize the knowledge about the inhibition of the SARS-CoV-2 and influenza virus propagation by targeting their RNA secondary structures.

## 2. SARS-CoV-2

SARS-CoV-2 is a typical member of the betacoronavirus family with a positive-sense, single-stranded RNA that includes a 5′ cap structure and a 3′ poly(A) tail [54]. The genome is around 29,900 nucleotides, which is larger than all the other RNA viruses. The first two-thirds of the genome encodes two overlapping polyproteins, ORF1a and ORF1ab, which are processed into 16 mature nonstructural proteins. They are associated with the replication/transcription complex, mediating the synthesis of genomic RNA (replication) and subgenomic mRNAs (transcription). The remaining part of the genome mainly codes four structural proteins: the spike (S), envelope (E), membrane (M), and nucleocapsid (N) [55].

**Figure 1:**
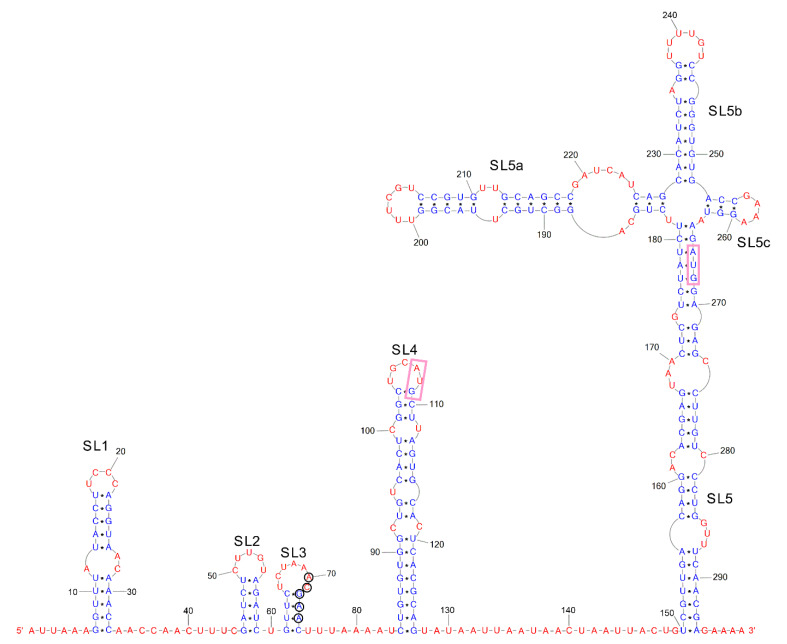
Scheme of SARS-CoV-2 5′UTR. The positions of the uAUG and the AUG codons are highlighted in pink boxes. Black circles indicate TRS sequences. Blue and red letter colors mark paired and unpaired nucleotides, respectively.

The secondary structure of 5′ and 3′UTR of SARS-CoV-2 is essential for virus propagation. The model of the 5ʹ-UTR, based on inline probing and enzymatic probing by RNase V1, was determined. This model is highly structured with plenty of accessible loops and bulges that fold into six hairpins, named SL1, SL2, SL3, SL4, SL4.5, and SL5 [56] (Figure 1). All the hairpins other than SL4.5 correspond with the bioinformatics secondary structure predictions for coronaviruses [57], including the SARS-CoV-2 [20]. This model is also in good agreement with the models obtained by mapping the whole SARS-CoV-2 genome in vitro [22] and in vivo [23,24,25,26].

The most variable hairpin among the SARS-CoV-2 variants is SL1 [56], which generally contains mismatches, bulges, and a high number of A–U and U–A base pairs. SL2 is conserved in all coronaviruses (CoVs) and usually possesses a pentaloop stacked on a five-base-pair stem. This hairpin participates in mouse hepatitis virus (MHV) translation and replication [58]. SL3 is conserved only in the subgroups of the beta and gammaCoVs [59] and possesses transcriptional regulatory sequences (TRSs) that are important in discontinuous transcription [12,57]. The SL4 is a relatively stable and long hairpin and possesses the start codon of an upstream opened reading frame [56]. SL5 is divided into SL5a, SL5b, and SL5c and contains an AUG codon. SL5a, SL5b, SL5c are found in all the coronavirus 5ʹ-UTRs that have been mapped so far [56,57,60,61,62]. 

**Figure 2:**
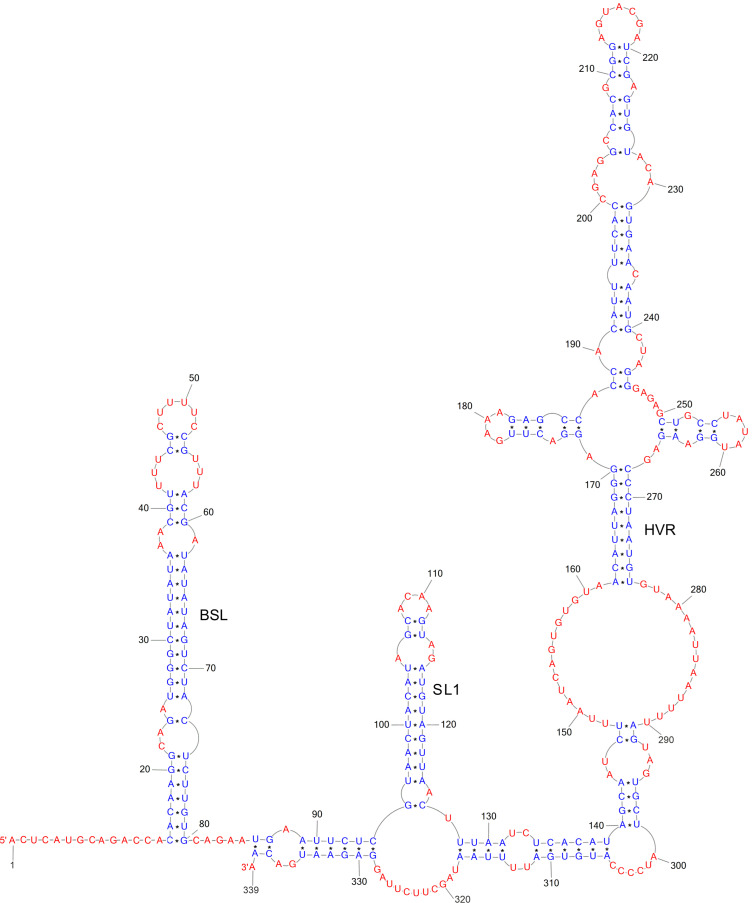
Scheme of SARS-CoV-2 3′UTR. Blue and red letter colors mark paired and unpaired nucleotides, respectively.

The 3′ UTR of the SARS-CoV-2 RNA contains various domains critical for the regulation of viral RNA synthesis and potential translation [20]. The studies suggest that the structure of the 3′ UTR of the RNA in the virus is identical to structures from cells previously infected with the virus [63] (Figure 2). This provides evidence that the secondary structure of the viral 3′UTR RNA is not influenced by the interaction between the viral RNA and the host or viral RNA-binding proteins. 

Three main secondary structures have been identified in the 3′ UTR: the bulged stem-loop (BSL), the SL1 loop, and the highly variable region (HVR) [64]. The results of bioinformatics analysis and reverse genetics suggest that a pseudoknot structure is formed at the base of the BSL and SL1 loop. It is proposed that the balance between the BSL and the pseudoknot is a molecular switch in SARS-CoV RNA transcription. The viral 3′ UTR contains the first binding site of the viral replication transcription complex (RTC) and many cis-acting regulatory elements necessary for viral proliferation [65]. The RNA structures derived from DMS-MaPseq in vivo have been shown to have a pseudoknot. In the case of the model coronavirus MHV, the pseudoknot conformation in the 3′ UTR structure was observed only with limited stability at 25 °C. At 37 °C, the MHV pseudoknot is transformed into an E-BSL conformer [66]. The literature data and the predictions of the pseudoknot structure based on the SHAPE-MaP map, the psoralen cross-linking, and the NMR spectrometry data suggest that the pseudoknot formation depends on ionic conditions and the presence of cellular proteins [22,24,26,67,68,69,70]. The existence of an equilibrium between the pseudoknot and the BSL in the 3′ UTR of RNA is supported by quantitative analysis of covariance (RF11065, Rfam database) [71]. It has been hypothesized that viral proteins may play a role in the formation of the pseudoknot conformation, meaning that the pseudoknot forms only transiently when the RTC binds to the 3′ UTR of the RNA in an ‘induced fit’ model [63]. In the model betacoronavirus MHV, the HVR region is not essential for viral RNA synthesis. However, some HVRs are highly conserved among betacoronaviruses, such as the stable S2M [20].

Next, a well-known structural RNA motif of SARS-CoV-2 is the frameshifting stimulation element (FSE) that occurs in ORF1ab, which comprises about two-thirds of the coronavirus genome [72,73]. ORF1a and ORF1b partially overlap, where ORF1b is located in the −1 reading frame relative to ORF1a [74]. The FSE takes part in the programmed regulating of a shift in the reading frame by one base in the 5′ direction; its role is due to its ability to pause the ribosome to initiate frameshifting, thus increasing the protein-coding capacity of the virus genome. The FSE possesses a conserved pseudoknot structure that has a three-stem architecture. Cryo-EM imaging and computational modeling revealed that the SARS-CoV-2 pseudoknot could adopt several different conformers [68,75,76,77,78].

### 2.1. siRNA as Potential Therapeutics

**Figure 3:**
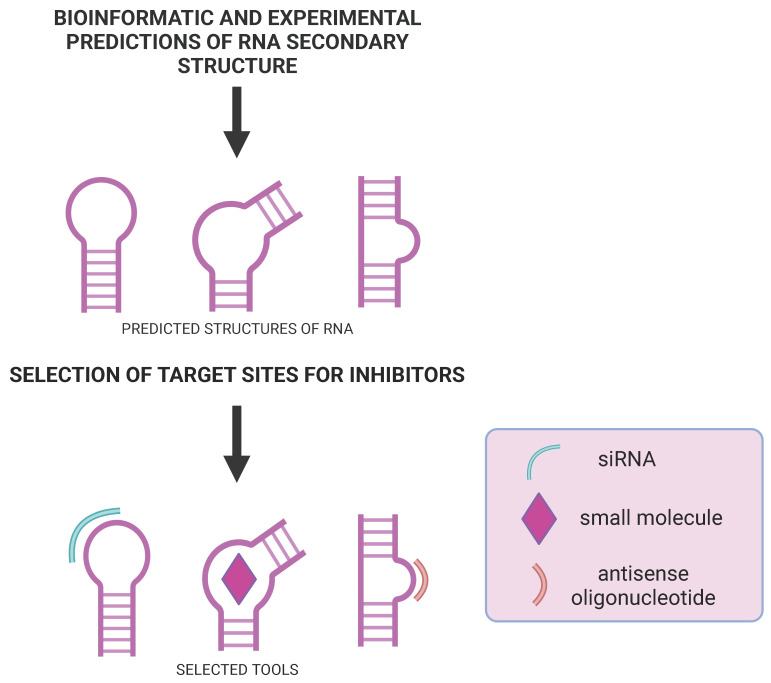
Scheme of the selection strategy of targeting sites of SARS-CoV-2 and influenza A virus RNA and choice of inhibition tools.

RNA interference is a cellular mechanism in which RNA is used to suppress gene expression [79] (Figure 3). The silencing pathway involves the cleavage of double-stranded RNA (dsRNA) into siRNA that is double-stranded with 21 to 25 base pairs and has two unpaired nucleotides at the 3′ ends [80]. 

Exogenous siRNAs are proven to be effective in regulating genes in different molecular studies and novel therapies. SiRNA molecules were also designed to inhibit viral infection by targeting and degrading viral RNA [81]. Several successful attempts to apply siRNAs were conducted against SARS-CoV-2 and the influenza virus [82,83]. 

### 2.2. siRNA Inhibitors Targeting SARS-CoV-2 RNA

To develop an anti-SARS-CoV-2 strategy using siRNA, Idris et al. designed 18 siRNAs targeting 5′UTR, RNA-dependent RNA polymerase (RdRp), and Helicase (Hel) genes [83]. After siRNA screening in Vero E6 cells and measuring the viral copy numbers by qRT-PCR assay, it was shown that three siRNAs demonstrated the most potent and dose-dependent repression of SARS-CoV-2 virus replication. Moreover, selected siRNAs were tested alone and in combination for the repression of SARS-CoV-2 virus replication. The authors found that mixtures of siRNAs exhibited the same viral knockdown as single siRNAs. One of the siRNAs, siUTR3, the targeted SL1 of 5′UTR, was subject to 2′ O-methyl chemical modification to increase its stability (Table 1). It was observed that the chemical modification into siUTR3 improved its stability in serum and extended the inhibition of the SARS-CoV-2 replication, although it is not as effective as unmodified siUTR3. The potential antiviral effect of selected siRNAs was tested in vivo using the K18-hACE2 mouse model of the COVID-19 disease infected with SARS-CoV-2 (Australian VIC1 strain). A liposome delivery platform (sLNPs) was used to deliver the siRNA. The sLNP-siRNA treatment resulted in less weight loss and a lower clinical score than in the control mice. After six days of treatment, it was also observed that the positive effect of siRNA was lost, suggesting that the repressive effect is transient. Finally, the research showed that the tested siRNAs significantly repressed SARS-CoV-2 replication in vitro and in vivo [83]. 

Niktab et al. designed six siRNAs specific to the SARS-CoV-2 mRNAs that targeted the membrane protein (M), an envelope protein (E), the spike protein (S), and the orf3a and orf1ab genes [88]. Before any testing of the efficacy of the siRNA, they minimized the off-target possibility of the siRNAs by aligning the RNA sequence of the SARS-CoV-2 virus with human RNAs. Similar sequences were excluded to ensure no influence of the siRNAs on the human mRNA. Moreover, they tested the effect of the siRNAs on the unaffected green monkey’s cells (Vero E6) without the SARS-CoV-2 virus and showed no cytotoxicity. To measure the efficacy of the siRNAs in the Vero cells infected by the SARS-CoV-2 virus from the serum of the affected patients, they used qRT-PCR. The paper did not specify the reference gene to normalize the Ct of the qRT-PCR, reporting only the Ct values themselves. The siRNA-treated infected cells have a higher Ct number and, therefore, a lower copy number of the virus mRNA. Based on the qRT-PCR analysis, it can be concluded that all the siRNA inhibitors used in the research inhibited the activity of the SARS-CoV-2 virus [88].

### 2.3. ASO as Potential Therapeutics

ASOs are single-stranded, chemically synthesized, short DNA or RNA that usually consist of 12–30 nucleotides (although shorter ASOs were also published). ASOs can modify the function of mRNA or other RNA, degrade RNA, or block gene expressions by complementary hybridization to an RNA target (Figure 3). Therefore, the sequence of ASOs is determined by the target sequence. The mechanism of RNA degradation by ASO, which is DNA or gapmer, occurs through the activity of the endonuclease RNase H, which induces RNA cleavage. ASOs not displaying RNase H enzyme activity are usually highly modified, and their mechanism of action may be steric blocking. Moreover, they can regulate RNA processing by interfering with the splicing machinery and exon skipping, promoting exon inclusion or cryptic splicing sites [106,107]. 

### 2.4. Antisense Oligonucleotide Modifications

Antisense oligonucleotides are chemically modified to reduce toxicity and to increase stability and protection from degrading agents. There are three generations of ASOs. The first generation of ASOs consists of phosphorothioate (PS), phosphoramidate (PA), and methyl phosphonate (MP) oligonucleotides. The first generation of ASOs is characterized by replacing the oxygen atoms from the phosphodiester bond with the sulfur, amine, or methyl group. This group of chemical modifications enhances ASOs cellular stability by making antisense oligonucleotides more resistant to nuclease and improves their membrane penetration [108].

The 2′-O-methyl (OMe) or 2′-O-methoxyethyl (MOE) oligonucleotides belong to the second generation of ASOs, which have a higher binding affinity for the target than PS. The antisense mechanism of second-generation ASOs involves the steric blockade of translation when targeting mRNA or other essential functions of targeted RNA. At the same time, recruitment and cleavage by RNAse H are impossible [106]. 

Finally, the three that are the most studied in the third-generation of ASOs are the locked nucleic acids (LNAs), peptide nucleic acids (PNA), and phorodiamidate morpholino oligomers (PMO). LNA possesses a methylene bridge connecting the 2′-oxygen with the 4′-carbon of the ribose. In PNA, the ribose-phosphate DNA backbone is replaced by the peptide-like N-(2-aminoethyl) glycine-linked nitrogenous bases of nucleic acids via a methyl carbonyl linker. PMO has the phosphodiester bond replaced by a phosphorodiamidate bond and the ribose by a morpholine moiety. With these chemical modifications, the antisense oligonucleotides form a stable hybrid with the target RNA. In addition to the main representatives of the modified ASOs, many laboratories have been working on new modifications, as well as different modifications that are used in one ASO [107,108].

### 2.5. ASO Inhibitors Targeting SARS-CoV-2 RNA

Zhu et al. designed gapmers with 3–4 LNA modifications at the 5′ and 3′ ends and a DNA-PS window of 9–10 nucleotides [84]. The ASOs targeted SL1 formed by the 5′ leader sequence of SARS-CoV-2 RNA. Huh-7 cells infected by the SARS-CoV-2 virus were used to test the gapmers. The viral RNA quantity was measured by qRT-PCR targeting the RNA of the nucleocapsid protein (N) and spike protein (S). Treatment with specific gapmers dramatically reduced the S and N gens levels. Out of many sequences of gapmers, 5′-ASO#26 (Table 1) displayed the highest level of the inhibition of viral replication [84].

Li et al. designed and synthesized antisense PMOs to find potential candidates for antisense therapeutics affecting SARS-CoV-2 replication [85]. The PMOs targeted the 5′ UTR TRS (hairpin SL3) of the genomic SARS-CoV-2 RNA (Table 1). SL3 is conserved among coronaviruses, making it a good target for antisense therapeutics. The antiviral properties of the designed PMOs were tested by measuring the virus level with the multiplicity of infection (MOI) in green monkey kidney Vero E6 cells. Moreover, a dose-dependent reduction in the SARS-CoV-2 RNA quantity was found [85].

Rosenke et al. investigated the anti-SARS-CoV-2 effect of the peptide-conjugated morpholino oligomers (PPMO) [86]. They designed and synthesized PPMOs targeting the 5′terminal region of the SARS-CoV-2 genome, the 5′ UTR TRS, and the translation initiation region (TIR) for ORF1a/b (Table 1). The qRT-PCR analysis showed that the PPMOs targeting the 5′ UTR TRS and the 5′terminal region highly inhibited the native SARS-CoV-2 virus growth in Vero E6 cells. However, the PPMO targeting TIR for ORF1a/b was less effective than the other ASOs [86].

Lulla et al. designed six LNA ASOs against the highly conserved structured s2m element from the 3′UTR of SARS-CoV-2 [109]. Moreover, they designed two other gapmers which have the same sequence but different polymer backbones. The first ASO was fully PS for improved resistance to nucleases. In the second gapmer, the backbone was mixed and contained DNA and LNA-PS. The research has shown that both modifications recruit RNase-H and lead to RNA degradation. To test the antiviral effects of the designed ASOs, the authors used astrovirus replicon-bearing SARS-CoV-2 s2m sequences transfected to Huh7.5.1 and HEK293T cells. The measured luciferase activity revealed inhibitions of the model replicon replication by three of the six gapmers [109].

Additionally, the Vero E6 cells were transfected with gapmers in three concentrations and infected by the SARS-CoV-2 virus. The high content screening (HCS) assay has demonstrated that gapmers 2 and 5 inhibited the SARS-CoV-2 replication the most, in a dose-dependent manner. In addition, at the highest tested concentration, these gapmers reduced virus replication by about 10% compared to the control [109].

Sun et al. used the in vivo click selective 2-hydroxyl acylation and profiling experiment (icSHAPE) technology to determine the secondary structure of the SARS-CoV-2 genome and, based on this finding, to design antivirals. [24] The cells of the human epithelial Caco-2 were transfected by the SARS-CoV-2-GFP-N construct in which the sequence encoding the N protein was replaced with GFP. At the same time, the oligonucleotides were transfected (Table 1). To determine the inhibitory effect of ASOs, fluorescence-activated cell sorting (FACS) and microscopy to detect the fraction of GFP-positive cells were used. Moreover, Huh7.5.1 cells were infected by SARS-CoV-2 to test the antiviral effect of the ASOs. The potential inhibitory properties of the ASOs were measured by qRT-PCR analysis of the RNA amount of the SARS-CoV-2 N gene. The ASOs tested in both cell systems demonstrated anti-viral properties and caused a decrease in the viral infection ratio compared to the negative controls [24].

Zhang et al. reported a 3D structure of SARS-CoV-2 FSE RNA by single-particle cryo-EM and tested the inhibition efficiency of several ASOs [76]. Before the cryo-EM results, to test the impact of the designed LNA ASOs targeting the 3′end of ORF1a, identified as FSE, they used a fully replicating SARS-CoV-2 luciferase reporter virus. The ASOs targeted FSE stem 2 and stem 3, deliberately avoiding stem 1, due to the predicted impossibility of antisense hybridization in the paired region of the secondary structure and the unfavorable predicted properties of the GC-rich LNAs required to target such regions. The inhibition effect of the ASOs was four times less compared to the EIDD-1931 (NHC, β-d-N4-hydroxycytidine, a ribonucleoside analog) used as a positive control. The cryo-EM results show that stem 1 has similar accessibilities as stem 2 and stem 3; therefore, the designed ASOs could demonstrate a considerable inhibitory effect compared to the ASOs targeting stem 2 and stem 3. The ASO targeting stem 1 presents better inhibitory activity against SARS-CoV-2 replication [76].

Effective LNA-modified oligonucleotides were designed based on a careful selection of the structural motif of the viral RNA of SARS-CoV-2 as a target. Two ASOs, LNA-12.8 and LNA-14.3, targeting the RNA motif of N and 5′UTR/ORF1ab, respectively, significantly reduced the viral titer of SARS-CoV-2. The experiments conducted on Syrian hamsters showed that LNA-12.8 prevents or mitigates SARS-CoV-2 transmission, diminishing virus titer in the lungs [87].

### 2.6. Small Molecules as Potential Therapeutics

Small molecules (SMs, ligands) are organic compounds that can bind tightly to specific structural motifs of RNAs and proteins due to hydrogen bonding, stacking, and electrostatic and hydrophobic interactions and should meet the Lipinski rules [110]. SMs can bind to RNAs and proteins very selectively and penetrate cellular membranes. The selective binding of SMs to RNA is possible because the target sites are structural elements (various types of mismatches, internal loops, and bulges) which determine the ligands’ solid binding (Figure 3). Because of the small size of ligands (molecular weight less than 500 Daltons), their large-scale chemical synthesis is relatively simple and cheap. Therefore SMs are attractive potential therapeutics against different diseases, including flu and COVID-19.

### 2.7. Small Molecule Inhibitors Targeting SARS-CoV-2 RNA

Although many scientists, especially those who determined or analyzed the RNA secondary structure of SARS-CoV-2, know that RNA motifs could be an excellent target for specific SMs and could lead to the development of a successful drug, there are not many published works on this subject so far [20,22,64]. Nevertheless, the research described below demonstrated a therapeutic potential for the targeting of the SARS-CoV-2 RNA structure with SMs.

Hanif et al. presented an approach to designing small molecules that target an attenuator hairpin (AH) of FSE within the genomic SARS-CoV-2 RNA [90]. The FSE includes, besides AH, a slippery site (SS) and a three-stemmed pseudoknot. The authors chose 3271 SMs from the RNA-focused library using their software: Inforna. Next, they used the microarrays approach (AbsorbArray) to screen the selected compounds and finally identified five SMs that bind to a short AH model. Among the recognized SMs, a small molecule, C5 (Table 2), interacted selectively in vitro with AH with a Kd = 11 nM. It was proved that C5 significantly decreased the frameshifting efficiency by 25 ± 1% in the frameshifting model (the Renilla-firefly luciferase reporter system) in HEK293T cells by stabilizing the AH structure. Two C5 analogs were synthetized: C5-RIBOTAC and C5-Chem-CLIP, to validate the binding site of C5 in cells when viral RNA is present. Both methods showed the specificity of the binding of the C5 targeted chimeras to the SARS-CoV-2 AH of the frameshifting element (Table 1). The authors also discovered that two more SMs (C1 and C3) influence frameshifting in the model system, enhancing the translation of the in-frame ORF; these are worth examining in more detail [90].

Sreeramulu et al. selected 15 structural motifs of SARS-CoV-2 genomic RNA that are thermodynamically stable in vitro, exist in vivo and ex vivo, and are conserved between SARS-CoV and SARS-CoV-2 [91]. The authors conducted an NMR-based screening of the DSI-poised library of 768 compounds. The authors reported that their finding was the first step of designing a leading compound but that they had already noticed promising SMs. Forty SMs binding to the 15 RNA motifs were selected; these belong to five classes in terms of their composition. One of the compounds, D01 (Table 2), was examined in more detail to distinguish the binding affinity and specificity. The authors concluded that the RNA motifs PK and 3_SL3base represent the most promising targets for continuation in the development of a lead compound. For example, D01 was bound with Kd = 6 µM to motifs PK and 3_s2m (Table 1). Unfortunately, the antiviral activity of any of found molecules has not yet been proven [91].

Zafferani et al. published dimethylamiloride (DMA) analogs targeting conserved motifs at the 5′-end of the SARS-CoV-2 genome and lowering the virus titer in a dose-dependent fashion in cells [92]. The SMs DMA-132, DMA-135, and DMA-155 (Table 2) reduced human coronavirus OC43 in Vero E6 cells ~1000-fold at a 100 µM concentration (Table 1). The same SMs at 10 and 50 µM inhibited the replication of wild-type SARS-CoV-2 in Vero E6 cells, as was monitored by a qRT-PCR assay and a TCID50 assay. The authors also used a dual-luciferase reporter assay that confirmed the antiviral activity and mechanism of action of the SMs. It was shown that the suppression of firefly luciferase (FLuc) expression required only the 5′-end of SARS-CoV-2 RNA, and DMA-155 reduced the FLuc signal to the highest extent (ca. 90%). The authors, based on NMR screening and the molecular docking of compounds to the predicted 3D structure of RNA motifs, postulated the structure-specific binding of found SMs to the motifs SL1, SL4, and SL5a of 5′-UTR, as well as SL6, located in the ORF1a of the SARS-CoV-2 genome [92].

An interesting approach was proposed by Zhao et al., searching for possible G-quadruplexes (G4) in SARS-CoV-2 RNA as targets for SMs [93]. Based on bioinformatics G4 prediction using the software QGRS-mapper and G4RNAscreener, the authors analyzed four putative G4-forming sequences (PQSs) of SARS-CoV-2 RNA. Several biophysical techniques confirmed the existence of G-quadruplexes for RG-1 and RG-2 in vitro, but only RG-1 G4 was thermodynamically stable. In the EGFP reporter gene system with the RG-1 G4 sequence (fragment of N gene) in HeLa cells, an SM pyridostatin derivative (PDP) (Table 2) was used to test the formation of G4 and the activity of PDP. PDP, the compound known to be specific for G4, reduced the protein level of SARS-CoV-2 N by stabilizing the RG-1 G4 structure (Table 1). This research showed a higher order structure element of G4 in the SARS-CoV-2 genome, which could be blocked by known and new SMs to gain virus proliferation inhibition [93].

Sun et al. applied the GFP/mCherry reporter system for the high-throughput screening of 4434 compounds to find SMs that impaired the programmed −1 ribosomal frameshift (−1 PRF) promoted by an RNA pseudoknot and in such a way disturbed the translation of the open reading frame 1b (ORF1b) [94]. Eight inhibitor candidates were selected and tested by using the luciferase-based PRF reporter assay. Finally, a pseudoknot binder, merafloxacin (Table 2), appeared to be a non-toxic, efficient inhibitor hampering SARS-CoV-2 replication in Vero E6 cells with an EC_50_ of 2.6 μM and an EC_90_ of 12 μM (Table 2). The authors quantified the abundance of nsp8 and nsp12 encoded by ORF1a and ORF1b, respectively. The relative amount of nsp12 and nsp8 was substantially reduced, proving the changes in the frameshifting [94]. 

The inhibition of SARS-CoV-2 replication by merafloxacin and a small molecule MTDB was shown as a result of searching for the pseudoknot binders as potential therapeutics [77]. From these two compounds, only merafloxacin in this independent research affected frameshifting, whereas the mechanism of action of MTDB turned out to be different (Table 1). 

The reporter system of SARS-CoV-2 −1 PRF, based on FLuc, was introduced by Chen et al. and used in the high-throughput screening of FDA-approved drugs [95]. The selected 52 compounds were then validated in a dual fluorescent protein reporter assay system. Finally, two SMs, (–)-Huperzine A and ivacaftor (Table 2), appeared to be active in the destruction of frameshifting in a model system. However, further research is required to confirm the effectiveness of these SMs as inhibitors of SARS-CoV-2 propagation [95].

### 2.8. The Other Methods

Other methods of inhibiting viral replication targeting RNA include, for example, the application of antisense PNA. PNA oligomers are single-stranded oligonucleotide analogs in which the sugar phosphate backbone is replaced by an N-aminoethylglycine-based polyamide unit that makes it uncharged. PNA binds to complementary RNA or DNA, forming high-affinity duplexes, or it can also form a triplex. In addition, an amide-based PNA backbone is more chemically, thermally, and enzymatically stable [89,111].

Li et al. designed and synthesized eight long PNAs covalently attached with cell-penetrating peptides (PPNAs) targeting the 5′UTR, 5′UTR TRS, and the polyprotein 1a/b translation start site (AUG) of the SARS-CoV-2 genome [89]. Subsequently, Vero E6 cells were infected by the SARS-CoV-2 virus in the presence of PPNAs. The determination of the viral RNA level was performed by qRT-PCR. The dose-dependent reduction in the viral RNA replication with increasing concentrations of PPNAs was observed. Curiously, the 5′UTR-3 PPNA decreased the SARS-CoV-2 titer by 75% compared to the control (Table 1). Moreover, almost complete inhibition was observed at a 10 μM concentration of 5′UTR-1 PPNA [89]. 

Su et al. designed several ASO chimeras that induced the inhibition of SARS-CoV-2 replication [96]. The chimeras were built with a 2′-OMe-modified phosphorothioate antisense oligonucleotide (ASO) and a 5′-phosphorylated 2′-5′ poly(A)4 (4A2-5). The designed way of action was to degrade the RNA of the envelope or spike proteins. The ASO part was for the sequence recognition of the viral RNA target, while the 4A2-5 was for the guided RNase L cleavage of the RNA. Using a pseudotyped SARS-CoV-2 infection model with GFP and FLuc reporters, the authors proved that the chimeras targeting the spike RNA had antiviral potential against the pseudotyped SARS-CoV-2 as well as its mutants and were better than the corresponding ASOs [96] (Table 1). 

## 3. Influenza A Virus

Influenza virus type A belongs to the Orthomyxoviridae family. The genome is constructed of RNA divided into eight segments that encode viral proteins: hemagglutinin (HA) and neuraminidase (NA); nucleoprotein (NP); two matrix proteins (M1, M2); the polymerase complex proteins, PB1, PB2, and PA; and four non-structural proteins. About 80% of the virus proteins are hemagglutinin and neuraminidase. Hemagglutinin is the protein that allows the virus to enter the host’s epithelial cells. Neuraminidase cleaves sialic acid residues, which allows the newly formed virions to be released [11,112,113]. The M1 protein is located inside the lipid envelope and is associated with vRNP (viral ribonucleoprotein) and the envelope, while the M2 protein acts as an ion channel [114]. The RNA is bound to multiple copies of the nucleoprotein and three subunits of viral RNA polymerase to form viral ribonucleoprotein (vRNP) complexes. Each segment consists of conserved, complementary 5′ and 3′ ends and one or more open reading frames. The 5′ and 3′ UTR ends of the vRNP are bound by viral RNA-dependent polymerase (RdRp) [115]. Internal base pairing within the influenza virus segments allows the formation of RNA secondary structures [16].

**Figure 4:**
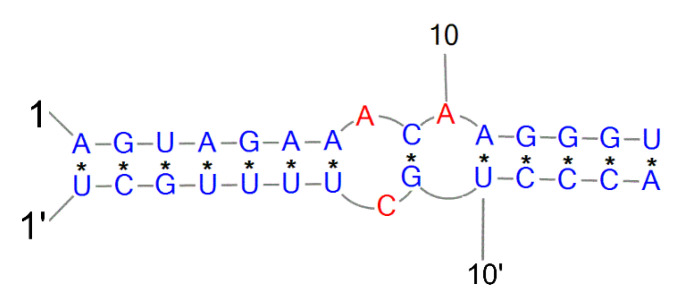
Secondary structure of influenza A virus panhandle. Blue and red letter colors mark paired and unpaired nucleotides, respectively.

The IAV promoter is never free in the life cycle of the influenza virus and is crucial in two mechanisms, transcription and replication [114]. The promoter region structure formed by the partial pairing of the 5′ and 3′ ends of each genomic segment [115] is a well-studied motif of the secondary structure of the influenza virus, called panhandle (Figure 4). This motif possesses a highly conserved sequence with incomplete complementarity, determining the circular nature of the RNP complexes. It includes 13 nucleotides from the 5′ end and 12 from the 3′ side, which is the binding site of the viral polymerase. Four possible variants of the promoter’s secondary structure have been proposed. The most characteristic of this region is the above-mentioned panhandle, which consists of a double helix containing non-canonical GU pairs, an internal single unpaired helix, and a unilateral bulge [41,42,43] The panhandle is dynamic in nature and undergoes remodeling depending on the stage of the virus replication cycle and the functions performed. The panhandle motif likely occurs when viral polymerase is absent. Another proposed model is the so-called fork, described as occurring at the time of transcription initiation [116,117]. The crystal structure obtained for the promoter-polymerase complex shows that the organization of the RNA ends resembles the corkscrew model [118]. The difference is the 3′ end, which is single-stranded and binds to the polymerase, giving rise to the hook model [119]. It is suggested that the switching of the viral polymerase function between transcription and replication occurs due to structural changes in the promoter region [118].

**Figure 5:**
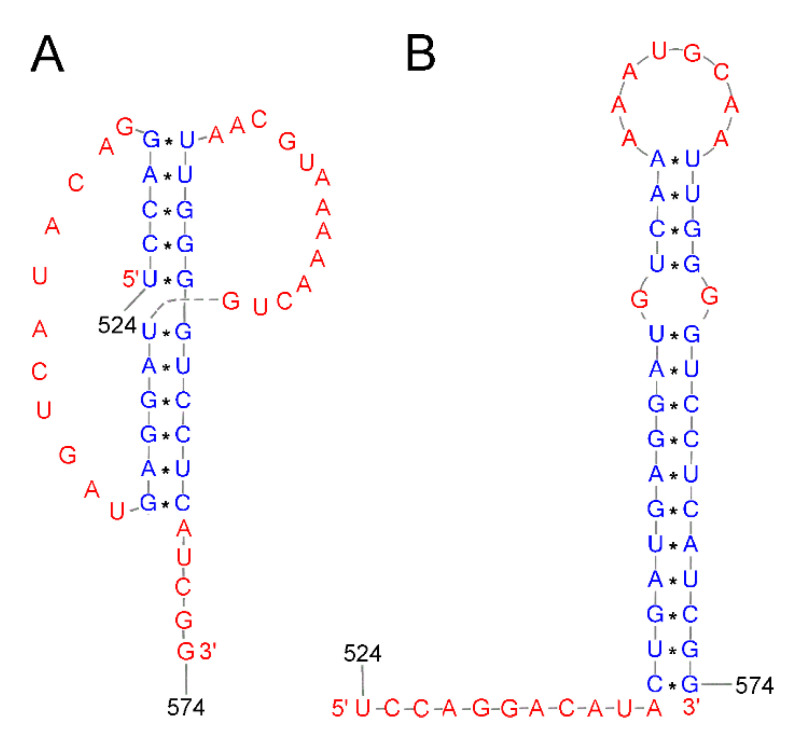
Pseudoknot (**A**) and hairpin (**B**) secondary structure of segment 8 (+) RNA of the IAV. Blue and red letter colors mark paired and unpaired nucleotides, respectively.

The secondary structure of (+) RNA plays a significant role in alternative splicing. Based on sequence alignment and chemical mapping experiments, a secondary structure was proposed for the 3′ (+) RNA part of segment 7 (vRNA7). Segment 7 encodes the M1 protein and the smaller M2 protein. The 3′ pre-mRNA region of segment 7 IAV can adopt two different structures: the pseudoknot and the hairpin. It has been proposed that this conformational change in structure may play a role in alternative splicing and affect the production of the M1 and M2 proteins [120,121,122]. These studies suggest that the splicing of segment 7 may be modulated by different splicing site accessibility [123,124] or splicing factor binding [125,126], and different conformation may be a mechanism to control the splicing of influenza genes [127]. Alternative pseudoknot and hairpin structures have also been identified in the splicing site 3′ of segment 8 (+) RNA of the influenza virus (Figure 5). Given the position of these structures in pre-mRNA, it has been suggested that the balance between them may have a function in splicing regulation [16,128]. Using bioinformatics tools, Gultiaev et al. predicted conserved secondary structures that could impose evolutionary constraints on HA vRNA. This analysis revealed that the structured domains in HA vRNA are subtype-specific. The researchers identified several domains that do not play a role in virus replication but may play a role in virus evolution and reassortment. Another analysis identified a functional pseudoknot structure in the NP vRNA packaging signal region [33,129]. 

Using bioinformatics tools, Kobayashi et al. also analyzed the structure of segment 7 of IAV RNA in the (+) and (−) strands [31]. They confirmed that the structure on both strands is similar and consists of a stem-loop (nucleotides 219–240) named SL3-10. They also showed that the same type of structure is found at positions 967–994 for both SL5-3B strands and with a second stem-loop in the nearby upstream region in the (+) strand at positions 950–964, named SL5-2. The mutations that changed the structures described above (SL3-10 and SL5-3B) disrupted virus replication, and SL3-10 mutants produced defective particles, which may lead to the conclusion that the secondary structure is crucial for packaging newly formed influenza viruses [31]. 

**Figure 6:**
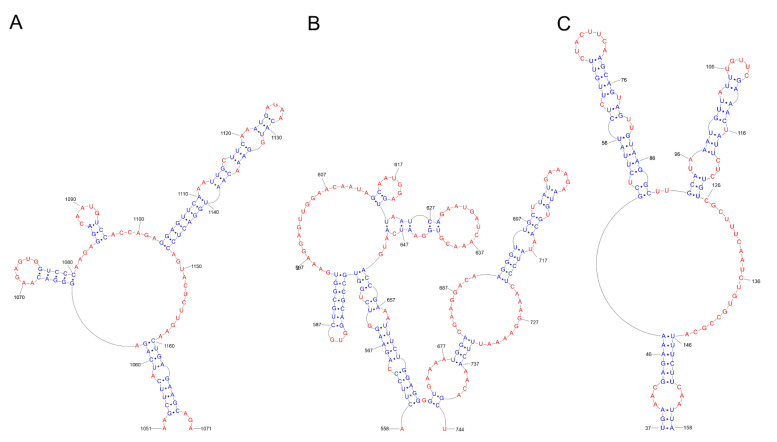
Secondary structure motifs of influenza A virus RNA that appeared to be a good target for inhibitors of virus propagation. (**A**)—M121 motif; (**B**)—selected motif of mRNA5; (**C**)—selected motif of vRNA8. Blue and red letter colors mark paired and unpaired nucleotides, respectively.

Jiang et al. examined the conserved RNA domain in the intron of influenza virus segment 8 (+) RNA, which encodes the NS1 protein and NEP protein—through alternative splicing [37]. Based on the experimental results, it was noted that the 81–148 region of segment 8 RNA folds into a hairpin structure. However, natural mutations may promote the reorganization of the structure, and the region exists with multiple conformations inside the cell. The researchers predicted that the conserved RNA domain in the intron of segment 8 might be necessary to regulate NS1 production. The function of this domain is not entirely clear, but its proximity to the 5′ splicing site may make it an enhancer/inhibitor of splicing [37]. The bioinformatics analysis of segment 5 RNA, which encodes the nucleoprotein, revealed a conserved structural motif in (+) RNA. Using in vitro and cell line experiments, Soszynska-Jozwiak et al. confirmed the predicted motif in (+) RNA segment 5 (nts 1051 to 1171), named the M121 motif [34] (Figure 6). The studied structure was also confirmed by probing the entire segment 5 (+) RNA of strain A/Vietnam/1203/2004 (H5N1). The conserved motif consists of three hairpins, one of which is particularly thermodynamically stable. To confirm the biological relevance of this structure, antisense oligonucleotides were used, with the target sites being two hairpins and a single-stranded linker that is likely involved in the tertiary interaction. The oligonucleotides that targeted the long hairpin and the inner loop were effective. These results suggest that the conserved M121 motif in the 5 (+) RNA segment may be a candidate for antiviral therapy [34]. 

The correlation between the function and the structure of the influenza virus RNA has been intensively studied for many years. The RNA secondary structures have been studied in vitro for segment 5: vRNA5 (strain A/Vietnam/1203/2004 (H5N1)), strain A/California/04/2009 (H1N1)), (+) RNA5 (strain A/California/04/2009 (H1N1)); segment 7: vRNA7 (strain A/Vietnam/1203/2004 (H5N1)); and segment 8: vRNA8 (strain A/Vietnam/1203/2004 (H5N1), strain A/California/04/2009 (H1N1)) [41,42,43,44,45] (Figure 6). Through the use of methods such as chemical mapping, isoenergetic microarrays, RNase H cleavage, assessment of base pairing probabilities, and conservation of canonical base pairs in strains, the complex nature of vRNA secondary structures has been revealed [130]. In addition to the known panhandle motif, other motifs have also been described [115]. Many hairpins have been identified in in vitro models that may have different functions. Several conserved hairpins have been revealed in the structure of the vRNA5, vRNA7, and vRNA8 of the influenza virus. The helical regions are usually separated by bulges and single-stranded loops, which can be functionally important natural interaction sites and become inhibitor attachment sites [31,41,42,43]. The structure of IAV mRNA in its native state in vivo during infection was reviewed by Simon et al. The research reveals that compared to the in vitro structure, IAV RNAs in vivo are less structured but exhibit specific local structures. Their functionality is indicated by the fact that the disruption by targeted mutagenesis resulted in impaired IAV replication capacity [48]. Soszynska-Jozwiak et al. determined the secondary structure of segment 5 (+) RNA of the influenza virus (strain A/California/04/2009 (H1N1)) [46]. Chemical mapping and free energy minimization were used to determine it. Two RNA constructs were studied: the full-length segment 5 (+) RNA and a shorter construct 5 (+) RNA containing only the open reading frame for NP. The second construct was analyzed to assess the effect of removing the partially complementary UTRs on the circularization of the molecule. The experiments revealed the presence of a hairpin in the 577–593 region, which also arises in the model with the deleted UTR and has been predicted by bioinformatics analyses [33,46]. In addition to the (+) RNA panhandle structure, there are two hairpins: one just upstream of the START codon and another containing the START codon, while the last two nucleotides of the START codon are paired to form the closing base pairs of the long hairpin. It is suggested that the involvement of the START codon in the hairpin structure could potentially regulate translation in influenza. The discovered motifs persist at both low and high temperatures. These results indicate that the stable and conserved motifs may be functionally important [46]. The secondary structure of the vRNA of segment 5 (strain A/Vietnam/1203/2004) was first determined by Michalak et al. Three domains were distinguished in the structure. The structural conservation of the motifs was shown to be moderately high for type A strains—87% of the base pairs were conserved. A characteristic motif of this structure (highly conserved among type A strains) is a 1527–1550 nt hairpin in the packaging signal. It is suggested that this motif is important for packaging. It appears that the bioinformatically predicted pseudoknot structure in the 90–130 region is not observed in vitro, which may suggest that it is strain-specific [33,41]. The authors compared the secondary structure of vRNA5 to (+) RNA5. Examples of similar structural motifs are 1341–1454 nt (107–227 nt in (+) RNA); 89–113 nt (1453–1477 nt in (+) RNA); 460–476 nt (1090–1106 nt in (+) RNA); 1243–1273 nt (293–323 nt in (+) RNA); 1472–1506 nt (60–94 nt in (+) RNA); and 1531–1545 nt (21 -35 nt in (+) RNA). These results complement previous studies and provide a global picture of the structure of the entire segment [41,46]. Using a similar approach, Michalak et al., in 2021, determined the secondary structure of influenza A virus segment 5 vRNA strain A/California/04/2009 (H1N1) to compare the structure of the vRNA5 of two distant strains: strain A/California/04/2009 (H1N1) and strain A/Vietnam/1203/2004 (H5N1). A comparison of the vRNA5 structure of both strains showed that the domain organization in the secondary structure model is preserved. In general, A/California/04/2009 (H1N1) vRNA5 does not contain the long helical fragments found in A/Vietnam/1203/2004 (H5N1), which consist of up to nine uninterrupted base pairs, such as 40–47/1309–1316, 327–334/622–629, and 843–850/1031–1038. Despite these differences, many motifs present in the secondary structure of A/Vietnam/1203/2004 (H5N1) vRNA5 exist in A/California/04/2009 (H1N1). The panhandle motifs and hairpin regions 87–115 nt, 975–987 nt, 1256–1265 nt, 1363–1375 nt, and 1527–1550 nt are present in both strains. Some hairpins differ only in the presence or absence of one base pair. Identifying common structural features is consistent with the reports stating that the RNA structure correlates with function. Stable, conserved RNA structures for different influenza virus strains are important for the virus replication cycle and are involved in key processes [44].

The secondary structure and structural motif analysis of the naked segment 7 vRNA of strain A/Vietnam/1203/2004 (H5N1) was proposed by Ruszkowska et al. [42]. The vRNA7 structure model consist of six domains. Domain I contains the panhandle motif. Domain IV and domain VI are the most structured domains in the model. The vRNA7 structure contains 20 hairpins with varying helix and loop lengths. Two hairpins have a very high degree of structure conservation (>99%): the hairpins spanning nucleotides 762–786 and 788–809. Some structural motifs of vRNA7 overlap with the vRNA genome packaging signals, suggesting their role in regulating viral folding. The researchers suggested that other structural motifs may be involved in binding proteins, recruiting editing enzymes to modify individual vRNA nucleotides (e.g., to avoid host immunity), and regulating transcription rates [42]. 

Lenartowicz et al. used a chemical mapping method combined with thermodynamics-based prediction and sequence/structure comparison to create a structural model of segment 8 vRNA (strain A/Vietnam/1203/2004) [43]. The secondary structure model agrees with the chemical mapping data and bioinformatics analysis of the conservative canonical base pairing of influenza A virus vRNA8. The structure consists of four domains. It has been shown that domains II-IV can fold independently, suggesting that they do not need to be simultaneously present at each stage of the virus life cycle. The base pairing of vRNA8 is highly conserved in influenza A viruses, with an average conservation of canonical base pairing of 82.6%. In domain III, single-stranded regions in three hairpins and a large bulge loop are present. They have low reactivity, which may indicate that they may form an alternative secondary structure. The vRNA8 coils into multiple probable helixes. These results show that segment 8, in its in vitro structure, contains structural motifs that are thermodynamically stable and conservative in influenza A virus strains [43]. Additional studies were carried out by Soszynska-Jozwiak et al. in 2021; they determined the secondary structure of segment 8 of the viral (−) RNA (vRNA) of the pandemic A/California/04/2009 (H1N1) IAV strain [45]. The structure was proposed based on classical chemical mapping and bioinformatics analysis. On average, the canonical base pairing in the vRNA8 of strain A/California/04/2009 (H1N1) is 89% conserved. The structure model is divided into four domains. Domain I contains the panhandle motif, which is involved in virus propagation [33]. Domains III and IV are the most reactive and contain hairpins and internal loops. The determined secondary structure of the vRNA8 of strain A/California/04/2009 (H1N1) was compared with the vRNA8 structure model of strain A/Vietnam/1203/2004 (H5N1), published in 2016 [43]. Many structural motifs in both strains are the same, such as in nucleotide regions 717–789, 1–28/850–890, 261–288, 293–347, 421–424/437–434, 175–179/468–464, 696–704/811–802, 710–713/796–793, and 98–102/122–113. The studied vRNA8 secondary structure allows the validation of the structural behavior of the A-type and strain-specific motifs. It appears that the secondary structure of vRNA8 for type A is largely universal, but several key differences are considered strain-specific [45].

Detailed analysis of the secondary structure of the viral RNA and information on the RNA interactions necessary for the viral replication cycle provide an understanding of the structure–function relationships and can help to predict regions of the influenza virus RNA to be considered in the design and development of potential antiviral tools.

### 3.1. siRNA Inhibitors Targeting Influenza A Virus RNA

The group of Zhang et al. investigated three siRNAs that targeted the polymerase (PA) gene of the avian influenza virus (A/Tiger/HarBin/01/2002 H5N1) for their ability to inhibit the replication of this virus [131]. In crystal structure analysis and mutagenesis studies, it was discovered that the N-terminal domain of PA contains an endonuclease active site and has therefore become an essential target for the design of new anti-influenza drugs [132,133]. Specific siRNAs were designed based on the N-terminal region of the PA gene, conserved among different subtypes and strains of the avian influenza virus. The results showed that PA-specific siRNAs inhibited the production of all the PA-specific mRNAs, vRNAs, and cRNAs in MDCK cells. Among the siRNAs tested, ps-PA496 showed the highest inhibitory activity, significantly reducing the viral RNA levels and protein expression (Table 1) The viral replication in the cells where ps-PA496 was delivered was 78-fold lower than in the controls. In the indirect immunofluorescence assay (IFA) assay, ps-PA496 caused a significant reduction in fluorescence. A mouse model of influenza virus infection was used to assess the inhibitory capacity of ps-PA496. A decrease in influenza virus titers was also observed in the lungs of mice injected with ps-PA496. These findings may have important implications for using siRNAs in preventing and treating influenza virus infection and understanding the mechanisms underlying influenza virus transcription and replication [97]. 

An interesting study was conducted by Piasecka et al., who designed siRNAs targeting structural motifs of segment 5 mRNA of influenza virus (coding NP) strain A/California/04/2009 (H1N1) [98]. Available regions for siRNAs were selected based on the secondary structure of the (+) RNA 5 segment. This approach led to effective inhibition of the virus proliferation and an understanding of the influence of RNA structural motifs on the replication cycle of the influenza virus. The researchers designed and tested siRNAs in MDCK cells, targeting 12 different regions of mRNA5 using differently chemically modified siRNAs. The siRNAs 613 and 682, with the highest antiviral potential, target the conserved regions of the secondary structure of influenza virus mRNA5: 613–631 and 682–700 (these regions exhibit 92.4% and 88.5% conservation, respectively) (Table 1) (Figure 6). SiRNA 613 partially overlaps with the binding site in hairpin loops. The second siRNA, 682, targeted a partially single-stranded region. These results show that the structural motif targeted by the siRNA may play a particularly important role in the viral life cycle. In addition, selected siRNAs were compared with antisense oligonucleotides, revealing that efficacy depends on target availability and antiviral strategy. Authors concluded that the best RNA target regions may be different for siRNAs and ASOs. In addition, selected siRNAs were tested with various modifications to improve siRNA inhibition. It was shown that the siRNAs modified with 2’-fluoro and triphosphate showed the highest antiviral activity. The present study identified regions of mRNA5 accessible to RNAi strategies, whose disruption significantly reduces influenza virus replication. Using knowledge of secondary RNA structures as additional criteria in siRNA design appears to be a promising therapeutic approach [98].

Jiang et al. used a novel approach to inhibit viral gene expression, protein synthesis, and the production of new virions [134]. The group used a swarm of DsiRNA molecules. DsiRNAs are chemically synthesized siRNAs between 25 and 27 nt in length that are substrates of the enzyme Dicer [135]. When introduced into mammalian cells, these molecules can be recognized and processed into shorter siRNAs of 21 bp in length by the endogenous Dicer, which facilitates siRNA loading into RISC and results in stronger RNAi induction than the canonical siRNAs [136]. The researchers evaluated the efficacy of DsiRNA swarming in preventing IAV infection in human primary monocyte-derived macrophages and dendritic cells. About 100 siRNAs were targeting the most conserved regions of the IAV genome. Conservative sequences were identified by matching sequences of avian/swine A/chicken/Jiangsu/cz1/2002, A/goose/Jilin/hb/2003, A/swine/Henan/wy/2004, A/wild duck/Hunan/211/2005, and A/avian/Hong Kong/0828/2007 and human A/Hong Kong/482/97, A/Vietnam/1194/2004, and A/Anhui/1/2005 viruses. Conserved sequences were identified in six segments of the H5N1 genome that encoded PB2, PB1, PA, NP, M, and NS proteins and analyzed using siVirus software. The siVirus program searches for functional regions in the viral genome. The replication of various IAV strains was inhibited by the pretransfection of cells with IAV-specific DsiRNA swarms. As much as a 7-fold inhibition of viral RNA production through the RNA interference pathway was observed. These findings provide opportunities for the use of DsiRNA in the prevention and therapy of IAV infection and warrant further studies in models of IAV infection in vivo [134].

### 3.2. ASO Inhibitors Targeting Influenza A Virus RNA

In 2008, the group of Duan et al. designed and tested an antisense oligonucleotide (IV-AS) that was specific for a conserved 5′ end sequence found in all eight segments of influenza A virus viral RNA [100]. The effect of IV-AS on influenza virus proliferation was monitored in vitro in MDCK cells and in vivo in a mouse model. The results indicated that IV-AS inhibited influenza A virus-induced cytopathic activity in MDCK cells. IV-AS was effective in preventing mouse death, reducing weight loss, and decreasing virus titers in the mouse lungs. The researchers suggested that the 5′ terminal conserved region of influenza A virus RNA segments may be significant in the study of potential drugs for preventing and controlling influenza virus infection [100]. In another study, Giannecchini used thiophosphate oligonucleotides (S-ON) derived from the packaging signals at the 3′ and 5′ ends of viral PB2 RNA, which were combined with liposomes and tested against the influenza virus in vitro [101]. A 15-mer S-ON replicating the 5′ end of viral PB2 and complementary to the 3′ end of its coding region showed inhibitory activity, probably because the packaging signal present at this end of PB2 plays an essential role in the efficient formation of infectious progeny virions (Table 1) The group assumes that a domain essential for the efficient assembly of influenza virions, the packaging signal located at the 5′ end of the PB2 segment, is a candidate for the development of novel antiviral compounds. This assumption is influenced by the fact that this region is highly conserved among influenza A viruses and plays an important role in proper virus assembly [101].

To inhibit viral proliferation, Lenartowicz et al. designed antisense oligonucleotides to target the internal regions of the viral RNA segment 8 (vRNA8) of influenza A/California/04/2009 (H1N1) [99]. Previously, the researchers determined the secondary structure of vRNA8 using experimental data, including chemical and microarray mapping, thermodynamics rules, and bioinformatic analysis to present regions accessible to ASOs across strains, beyond the conserved ends. All the ASOs targeted single-stranded and accessible regions of the vRNA8 structure. The ASOs were predominantly 2′-O-methyl RNA, and half contained modified LNA nucleotides to stabilize binding to the target sequence. Seven of the ten ASOs tested inhibited virus growth at least threefold in MDCK cells. The most potent inhibitory potential was demonstrated by ASOs 68-11L, 187-14L, and 404-14L, which reduced the virus titer more than 10-fold (Table 1). ASO 68-11L and 404-14L mainly targeted single-stranded regions in the hairpin loop, while ASO 187-14L targeted two internal bulges, a partially single-stranded region, and a fragment of the double helix [99]. This approach is promising as antiviral activity was observed when targeting genomic sequences outside the panhandle region, which was previously thought to be the only vRNA region available for targeting with ASO [100,101]. Michalak et al. used a similar approach, where the secondary structure of vRNA segment 5 of A/Vietnam/1203/2004 was experimentally determined. Summarizing the bioinformatics data and structural mapping revealed the secondary structure and previously unknown internal motifs and single-stranded regions. In the proposed secondary structure model, there are motifs previously predicted by Gultyaev et al. that were likely in both (−) and (+) strands as “mirror structures” [33]. The structure proposed by the researchers is 87% conserved in influenza A virus sequences. The designed ASOs confirmed the presence of internal single-stranded regions. The study used modified RNA oligonucleotides (2′OMeRNA and 2′OMeRNA-LNA), ensuring their suitability for low-dose oligonucleotide antisense strategies. They proved that five ASOs inhibited viral replication in MDCK cells. The most potent oligonucleotides reduced viral titers by 90%; so, these results point to structural regions universal to influenza type A that are important for virus function. The most effective inhibitory oligonucleotides were 883-11L, 474-21M, and 1253-13M. The 883-11L oligonucleotide targeted the single-stranded region, the 474-21M oligonucleotides targeted the two small hairpins, and 1253-13M targeted the partially single-stranded and hairpin region. This study was critical because it indicated the possibility of using antisense strategies targeting vRNA5 based on its structure and secondary structure conserved motifs, which could become powerful alternative therapies against influenza [41].

Determining the secondary structure preceding the design of sites available for the ASO is also essential for (+) RNA. Soszynska-Jozwiak et al. determined the secondary structure of segment 5 of (+) RNA of the influenza virus [46]. Chemical mapping and thermodynamic energy minimization were used to determine it. Sequence analysis showed that the secondary structure of RNA segment 5 (+) is conserved between influenza type A strains. At the same time, microarray mapping and RNase H cleavage provided the opportunity to identify target sites for the ASOs in the tested structure. Nine of the twenty-one ASOs tested showed inhibition of influenza virus replication. The most effective ASO was ASO 727A, which targeted the long inner loop and inhibited influenza A virus propagation 7-fold (Table 1). The group reports that the most effective ASOs targeted the inner loop motifs or hairpin loops, indicating that these two domains are good targets for oligonucleotides and may be a target for antiviral therapy [46].

The influenza virus mutates rapidly and can develop resistance to antiviral drugs. The group of Hagey et al. characterized the loop-stalk structure of PSL2 RNA, which serves as a packaging signal for the PB2 segment [87]. First, the secondary RNA structure in PB2 that mediates the packaging was mapped. Then, the role of this structure in the viral life cycle and IAV pathogenesis in vivo was genetically validated. The PSL2 motif mediates viral packaging in vitro, and it was found that the stem-loop structure of PSL2 was necessary for viral packaging in the cellular environment. The ASO with modified LNA bases was designed and targeted to PSL2 (Table 1). The ASOs designed to disrupt the structure of PSL2 dramatically inhibited the IAV of various strains and subtypes, which often show susceptibility to drug resistance. RNase-H-activated ASO LNA9, targeting the partially double-stranded region, had the most robust antiviral properties. It appeared that the ASO targeting PSL2 also enabled mice to develop resistance. This approach may be used against vaccine-resistant virus strains in the future [87].

### 3.3. Small Molecule Inhibitors Targeting IAV RNA

So far, few attempts have found SMs binding to influenza RNA as future potential therapeutics. An elegant approach was proposed by Varani, Pellecchia, and coauthors in two of their papers [102,103]. They selected as an object a panhandle structure of IAV vRNA. The authors conducted NMR-based fragment screening of 4279 compounds, allowing them to find binding scaffolds that interact with the panhandle. They identified 6,7-dimethoxy-2-(1-piperazinyl)-4-quinazolinamine (DPQ) (Table 2), which bound to the panhandle with a Kd of 50.5 ± 9 µM (Table 1). The NMR structure of the complex DPQ/RNA panhandle model revealed that DPQ fits tightly into the major groove of the internal loop at the (A–A)–U loop. Additionally, DPQ binding broadens the major groove and the end of the helix compared to the RNA without a ligand. For determination of the inhibitory activity of DPQ, they used an FDA-based CPE assay to estimate the CC_50_ and EC_50_. The results showed that DPQ was non-toxic to cells at concentrations up to 500 µM. It inhibited the replication of two different strains of influenza A and one type B, but at a lower degree than amantadine, oseltamivir, or ribavirin. An additional assay as a plaque reduction assay showed that 56 to 500 µM of DPQ inhibited virus growth. The experiments with a modified virus, WSN-Ren luciferase, in modified MDCK-HA cells confirmed the potential of DPQ (measured in this system IC_50_ was equal 435 µM) as well as two more selected compounds as influenza inhibitors. Taking together the collected observation, the DPQ binding changes the panhandle structure and such a method could interfere with the binding of RdRp and consequently disturb replication [102].

Following these studies, the authors designed and tested DPQ analogs to facilitate the SM binding affinity and pharmacological properties based on a DPQ/RNA complex structure [103]. The 15 analogs containing modified and/or extended secondary amine on the piperazine were examined. This approach was successful, and a group of compounds with better anti-influenza properties were found and characterized by IC_50_, CC_50_, IC_50_/CC_50_, and Kd values using cytotoxicity assays, the WSN-Ren luciferase/MDCK-HA assay, real-time PCR, and NMR spectroscopy. Compounds 7 and 10 (Table 2) possessed excellent IC_50_/CC_50_ ratios < 0.14, displaying high inhibitory activity and low cytotoxicity, whereas SMs 6 and 8 (Table 2) had a IC_50_/CC_50_ ratio < 0.18, which is still is suitable for the potential drugs [103] (Table 1). 

### 3.4. The Other Methods

In light of the continued variability of IAV and its increasing resistance to the existing drugs and vaccines, finding target sites in IAV RNA that are sequentially or structurally conserved and less susceptible to drug resistance is a fundamental issue. Here are some examples of tools other than siRNA and ASO targeting influenza A virus RNA that may become useful in the design of antiviral therapies.

A novel approach was taken by Kesy et al., who used a promising tool to complement traditional methods of influenza virus inhibition [104]. The group used a chemically modified short dbPNA (dsRNA-binding PNA) oligomer as an inhibitory tool against the influenza virus, targeting the panhandle structure (Table 1). Cellular studies have shown that the panhandle structure targeting dbPNA-neamine conjugate IR-1b is a potent antiviral agent against various subtypes (H1, H5, and H7) of influenza A viruses. The modified dsPNA was shown to bind selectively to the dsRNA region of the panhandle motif. It was observed that the dbPNA-neamine conjugate resulted in reduced viral replication. Interestingly, dbPNA inhibits innate immune receptor RIG-I binding to the panhandle structure, and thus RIG-I ATPase activity. These discoveries will allow the development of new therapeutic agents against the influenza virus [104].

Interesting observations were made by Czapik et al., who proposed new ribozyme hammerhead variants targeting conserved structural motifs in RNA segment 5 (+) of influenza virus strain A/California/04/2009 (H1N1) [105]. The variants were enriched with structural and chemical modifications; an additional hairpin motif was introduced as well as an attempt was made to select ribozyme target pairs with sequence features that enable the potential formation of the trans-Hoogsteen interactions. The highest statistically significant inhibitory effect on IAV replication was obtained for the ribozymes named RZ6A and RZ6C, which reduced replication by 37.4% and 30.2%, respectively. The ribozymes targeted the (+) RNA5 615–704 region of the IAV. The ribozymes showing the most significant inhibitory activity against the influenza virus, such as RZ6A and RZ6C, were selected to prepare chimeric shRNA ribozyme constructs (named sh613RZ6A and sh613RZ6C). Infected MDCK cells were treated with plasmids containing the shRNA-ribozyme constructs, resulting in the inhibition of viral replication (Table 1). Moreover, qRT-PCR analysis showed a significant decrease in viral RNA following the use of plasmids with ribozyme constructs enriched with the hairpin structure. The lowest viral titer was achieved with the use of the chimeric sh613RZ6C construct [105]. This use of ribozymes is promising, as it may allow the future development of constructs targeting important structural domains to achieve significant antiviral effects.

A different approach was taken by Peng et al., who studied approximately 300 miRNAs derived from human and mouse epithelial cells [137]. They analyzed the mechanisms underlying the function of the miRNAs as an inhibitory factor for the expression and replication of human influenza virus strain H3N2 and A/Puerto Rico/8/1934 (PR8; H1N1). The role of five highly expressed suppressive miRNAs targeting viral RNA or supporting host factors was experimentally investigated. Of the miRNAs tested, five were shown to have an inhibitory effect. Hsa-mir-127-3p, hsa-mir-486-5p, hsa-mir-593-5p, and mmu-mir-487b-5p were found to target at least one viral gene segment of both seasonal influenza H3N2 and attenuated PR8 virus. The fifth miRNA inhibited viral replication by targeting the ATP6V1A supporting factor. These studies suggest that miRNAs regulate different stages of the viral life cycle by targeting viral RNA or host factors [137]. In another study, hsa-miR-323-5p, hsa-miR-491-5p, and hsa-miR-654-5p were shown to directly bind to viral RNA PB1, exerting an inhibitory effect on IAV replication in MDCK cells. This work raises interest in the delivering of exogenous miRNAs as therapeutic agents for antiviral therapy [138]. The study of miRNA molecules that directly target conserved viral RNA sequences could lead to the discovery of new therapeutic agents against influenza virus infection.

## 4. Conclusions

The development of bioinformatic and experimental methods for determining the RNA secondary structure allows for the creation of effective methods of inhibiting the multiplication of the SARS-CoV-2 and influenza A viruses. To date, the presence of conserved RNA structural motifs in SARS-CoV-2 and IAV have been identified. ASOs, siRNAs, SMs, miRNAs, and other tools were tested and effectively inhibited SARS-CoV-2 and influenza A virus propagation in cells, and some of them were tested in mice [64,139]. Similar attempts to use RNA-targeting strategies were conducted against both viruses. The authors proposed interesting approaches, but not all the inhibitors were validated against the native virus, especially in case of the SARS-CoV-2 research. Structural RNA motifs have proven to be good targets for future therapy development. In focus were regulatory motifs, motifs responsible for certain protein binding, motifs which structural changes are important in the viral cycle, and structural motifs with unknown functions but which are evolutionarily conserved both in coding and non-coding viral RNA. From the presented studies herein, it can be concluded that some inhibitory tools are more suitable for certain RNA targets than others. The example is the higher effectiveness of siRNA targeting the same motif of mRNA5 IAV than ASO. The accessibility of the target as well as the mechanism of action of the inhibitor seems to be a key for success. 

A direct and fair comparison between published inhibitors is not possible because the authors used different systems for validations (different cells, animals, virus strains, reporters, and ways of measurement) in different conditions. However, some general tendencies can be seen and may be a typical feature of a given strategy. Oligonucleotide tools (ASO and siRNA) were successful in a low dosage, even a nanomolar range (the best was 8–50 nM) compared to small molecules, which were used in the µmolar range (the best was 20–50 µM) to achieve maximal effect in cells. In the case of ASO, the design by the authors of the modification of the parent ASO did not always improve it. Clearly, in the field of ASO design a systematic knowledge of the influence of different modifications is lacking. The effectiveness of oligonucleotide is also very dependent on the precise target site, and step-by step screening of some regions gave a better chance of finding the best inhibitors. The discovery of inhibitory SM is mostly based on different types of screening. SM could be specific but proving its non-toxicity and the identification of binding sites are usually very challenging. In addition to target RNA, SM could theoretically bind to different nucleic acids and proteins and such phenomena need to be excluded. The most advanced method of SM binding site determination required its chemical modification, which could diminish SM performance. Despite such difficulties, small molecules remain promising RNA-targeting tools.

All kind of inhibitors need to be successfully delivered. Different types of delivery of oligonucleotides were used in the treatment of IAV and the SARS-CoV-2 infection and the knowledge in this field is growing. The delivery of small molecules is not as universal as for nucleic acids, but on the other hand, small molecules do not usually need additional vehicles for cells penetration. One of the problems is solubility in water, and very often, a toxic DMSO needs to be added. The other difficulty when testing SM is confirmation of the proper distribution in the cells. The good news is that in the case of future COVID-19 or flu treatment a simple intranasal delivery of both the oligonucleotide and the small molecule inhibitor was successful in mice.

In the usually proposed pathway of inhibitor design and validation, the jump from in vitro to in cells and further in mouse and human research often eliminates promising compounds. On the other hand, it is important to exclude in simpler systems candidates that not working in the designed way. Moreover, it is essential in modern medicine to know a drug’s mechanism of action. Therefore, the research presenting inhibitor specificity for selected targets and proving the method of activity is very valuable. 

Chimeras of different tools were also proposed against IAV and SARS-CoV-2, but such compounds are complicated and rarely fruitful. From the successful stories of oligonucleotides or small molecule drugs, we can learn that a relatively simple compound can be a good drug when the target, rational modifications, and delivery are all considered and optimally used. The perfect drug needs to be specific, stable in the cellular environment, effectively delivered, active in a low dosage (to minimize possible side effects and reduce price) with no or minimal off-target effect. Additionally, in case of the respiratory tract viruses, a relatively high inhibition is required to obtain a substantially reduced viral titer. Only in that case can an improvement in the clinical condition of the patients during a rapid course of illness be achieved.

## 5. Perspectives

Because RNA viruses are characterized by high mutation rates [140], there is growing scientific interest in the host factors that support viral infection. To achieve replication, RNA viruses interact with many cellular molecules via protein–protein, RNA–protein, protein–lipid, and RNA–long noncoding RNA interactions [141,142,143]. Böttcher-Friebertshäuser et al. used peptide-conjugated phosphorodiamide morpholino oligomers (PPMOs). They were single-stranded-DNA-like antisense agents that can sterically block RNAs. The PPMOs targeted the pre-mRNA or mRNA regions of the TMPRSS2 protease that cleaves hemagglutinin (HA) and the lymphocyte activation-3 gene. Altered splicing of TMPRSS2 mRNA by PPMOs (T-ex5) resulted in the expression of a truncated and inactive form of TMPRSS2, which prevented HA cleavage in human seasonal and pandemic influenza viruses and reduced viral titers. McCollum et al. demonstrated that microRNA-2392 circulates in patients infected by SARS-CoV-2, and its quantity increases depending on the viral load.

The increased concentration of microRNA-2392 during COVID-19 may be used as an effective biomarker. Therefore, McCollum et al. developed Nanoligomer SBCoV207 with a specific PNA component designed to target microRNA-2392 to treat COVID-19. To test viral replication inhibition with this tool, the African green monkey’s kidney epithelial cells (Vero E6 cells) and human lung epithelial cells expressing human ACE2 (A549-hACE2) were infected with SARS-CoV-2 and treated with Nanoligomer SBCoV207. The viral replication inhibition was measured by immunofluorescence assay for the SARS-CoV-2 nucleocapsid protein (N) and showed a significant reduction in virus titer compared with the control. Moreover, the viral mRNA of TRS-N was measured by qRT-PCR and indicated that the viral mRNA abundance was reduced approximately 10-fold [144]. Targeting elements other than the RNA of SARS-CoV-2 and the influenza virus is a novel antiviral strategy and may additionally limit the emergence of virus mutant escape. Host cell factors involved in infection are rarely considered in the design of anti-influenza therapies due to possible toxicity [145]. The above studies suggest that not only targeting virus RNAs but also targeting the human factors that promote virus proliferation can inhibit SARS-CoV-2 and influenza virus replication and be used to design antiviral therapies. A better understanding of the molecular mechanisms of virus–host factor interactions will provide insights for the development of innovative, broad-spectrum antiviral therapy against viral infections.

## Figures and Tables

**Table 1:** RNA motifs of SARS-CoV-2 and IAV as a target of viral inhibitors. Selected inhibitors and theirs effect are listed. * the effect of inhibitors cannot be compared with each other because of different systems used in evaluations by different authors. Numbers listed are as authors wrote or, if not precise in text, are deducted from the publication’s figures. For better uniformity of presented results, it was decided to show the data from qRT-PCR of cell experiments (if conducted) for the best tested concentration of inhibitor. If qRT-PCR was not conducted, another measurement was presented for the best tested concentration. Main animal experiment results were additionally listed. The method of measurement was indicated.

**SARS-CoV-2**					
**Name**	**Region (nt)**	**Target**	Effect *	**Type of Inhibitor**	**Reference**
5′-ASO#26	677–692	5′ UTR	inhibition: reduced by 90–95% of viral mRNA level (qRT-PCR)	ASO	[84]
TRS-PMO	70–75	5′ UTR TRS loop	inhibition: reduced by 20% of viral RNA level (qRT-PCR)	ASO	[85]
5′END-1 5′END-2 TRS-1 TRS-2 AUG	1–24 5–29 59–82 53–77 251–275	5′UTR TRS loop	inhibitions: Ct increase (qRT-PCR)	ASO	[86]
ASO-ORF1ab-6449 ASO-ORF1ab-9456 ASO-N-29502	6449–6498 9456–9525 29,502–29,541	ORF1ab N gene	inhibitions: reduced by 40–50% of viral RNA level (qRT-PCR)	ASO	[24]
LNA-12.8 LNA-14.3	28,743–28,759 260–275	N gene 5′UTR/ORF1ab	inhibition: reduced by 3.0, 3.2 log_10_ virus titer (plaque assay); reduced by 2.9–5.0 log_10_ (LNA-12.8) of virus titer in the Syrian hamster lungs	ASO	[87]
siRNA mix	23,054–23,076 8487–8505 2–20 25,529–25,548 27,000–27,019 26,359–26,378	S gene ORF1ab ORF1ab M gene E gene	inhibition: Ct increase (qRT-PCR)	siRNA	[88]
siUTR3 siModUTR3 siHel1 siUC7	10–30 10–30 17,830–17,850 15,836–15,856	5′ UTR helicase gene	inhibition: reduced by 75–100%, 25–100%, 75%, 83% of viral titer (plaque assay); reduced by 1–1.6 log_10_ (siUC7), 1–1.6 log_10_ (siHel1) mice lung titer	siRNA	[83]
5′UTR-1 5′UTR-3 AUG	21–39 248–265 254–271	5′ UTR TRS loop 5′ UTR	inhibition: reduced by 99–100% (IC_50_ = 0.8 µM), 75% (IC_50_ = 6.8 µM) and 23% of viral RNA level (qRT-PCR)	PPNA	[89]
C5	13,433–13,457	AH, ORF1a	decreased frameshifting efficiency of construct: reduced by 25% of viral RNA level (RT-qPCR)	SM	[90]
D01	13,475–13,542 29,619–29,870	PK in ORF1b 3_SL3base in ORF10/3′UTR	binding to target: K_d_ = 6.0 (fluorescence-based titration)	SM	[91]
DMA-132 DMA-135 DMA-155	7–33 77–136 183–227 302–343	SL1 in 5′UTR SL4 in 5′UTR SL5A in 5′UTR SL6 in ORF1a	reduced by 50%, 50%, and 90% of reporter construct expression (dual-luciferase reporter assay)	SM	[92]
PDP	28,903–28,917	RG-1 G4 in gene N	reduced by 40–90% of protein expression of reporter construct (flow cytometry, IVT assay)	SM	[93]
merafloxacin	13,475–13,540	PK in ORF1b	inhibition: EC_50_ = 2.6 μM (plaque assay)	SM	[94] [77]
(-)-Huperzine A ivacaftor	13,433–13,540	-1 PRF in ORF1a/ORF1b	reduction in –1 PRF: lowering by 96% of luciferase level (PRF luciferase assay)	SM	[95]
S4 ASO-4A2-5 S5 ASO-4A2-5 S6 ASO-4A2-5	23,032–23,046 23,080–23,094 23,270–23,284	S gene	reduction in expression: lowering by 87%, 73%, 69% of luciferase level (luciferase reporter assay)	chimera	[96]
**Influenza A Virus**					
**Name**	**Region (nt)**	**Target**	Effect *	**Type of inhibitor**	**Reference**
ps-PA496	163- 178	segment 3 (+) RNA	inhibition: 78-fold lower viral titer (immunofluorescence assay); decrease by 0.83 log_10_ TCID50/g of viral titer in mice lungs	siRNA	[97]
682 682′	682–700	segment 5 (+) RNA	inhibition: reduced by 85.6%, 78.0% of viral RNA copy number (qRT-PCR)	siRNA	[98]
613 613′ 613-sF1 613-sF2	613–631	segment 5 (+) RNA	inhibition: reduced by 84.5%, 85.3%, 88.3%, and 88.4% of viral RNA copy number (qRT-PCR)	siRNA	[98]
68-11L 404-14L 187-14L	63–73 398–411 181–194	segment 8 (−) RNA	inhibition: 20-fold, 25-fold, and 16-fold decrease in virus titer (immunofocus assay)	ASO	[99]
474-21M 883-11L 1253-13M	465–485 878–888 1248–1260	segment 5 (−) RNA	inhibition: reduced by 64%, 88%, and 48% of viral RNA copy number (qRT-PCR)	ASO	[41]
727A 3A 2A 400A 615A 640A	722–732 1141–1156 1146–1156 398–405 609–621 628–644	segment 5 (+) RNA	inhibition: reduced by 87%, 79%, 72%, 71%, 70%, and 64% of viral RNA copy number (qRT-PCR)	ASO	[46]
IV-AS	1–13	5′-terminal conserved region of the eight influenza (−) RNA	inhibition: EC_50_ = 2.42–3.95 µM (CPE assay); decrease by 4.49 log_10_ TCID_50_/g of viral titer in mice lungs	ASO	[100]
5-15b	2279–2294	5′ end of the segment 1 (−) RNA	inhibition: reduction by 3.4 log_10_ of viral titer (TCID_50_/_mL_)	ASO	[101]
LNA9 LNA8 LNA7	73–88	segment 1 (−) RNA	inhibition: reduction by 4.5, 3.5, and 3.8 log_10_ of virus titer (plaque assay); reduced by >2.5 log_10_ (LNA89) of virus titer in the mice lungs (CCID/mL)	ASO	[87]
DPQ	1–15/1′14′	panhandle in 5′UTR/3′UTR (−) RNA	inhibition: EC_50_ = 71.6–275.5 µM (CPE assay)	SM	[102]
DPQ analog 7 DPQ analog 10	1–15/1′14′	panhandle in 5′UTR/3′UTR (−) RNA	reduction in expression: IC_50_ = 33.89 µM IC_50_ = 34.18 µM (reporter assay)	SM	[103]
IR-1b	1–15/1′14′	panhandle in 5′UTR/3′UTR (−) RNA	inhibition: reduction by 70% of viral RNA copies (qRT-PCR)	dbPNA	[104]
RZ6A RZ6C	615–704	segment 5 (+) RNA	inhibition: reduction by 37.4% and 30.2% of relative viral RNA level (qRT-PCR)	ribozyme	[105]
sh613RZ6A sh613RZ6C	615–704	segment 5 (+) RNA	inhibition: reduction by 85.9% and 80.0% of relative viral RNA level (qRT-PCR)	chimeric shRNA ribozyme construct	[105]

**Table 2:** Structures of most potent SARS-CoV-2 and IAV SM inhibitors.

**Structures of Selected SARS-CoV-2 SM Inhibitors**	
C5 [90]	D01 [91]
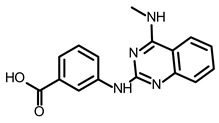	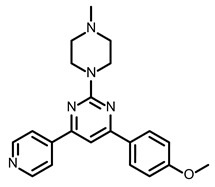
DMA-132 [92]	DMA-135 [92]
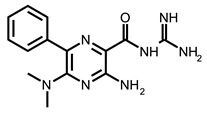	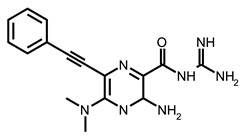
DMA-155 [92]	PDP [93]
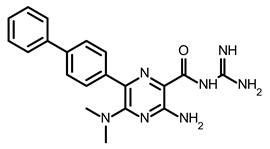	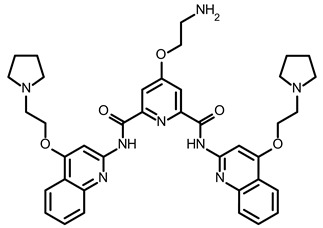
Merafloxin [94]	Huperzine A [95]
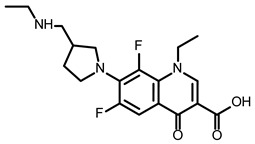	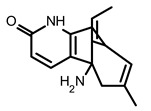
Invacavtor [95]	
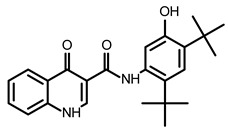	
**Structures of selected IAV SMs inhibitors**	
DPQ [102]	Compound 6 [103]
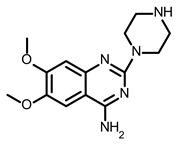	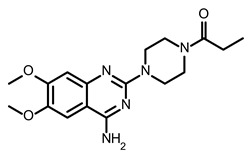
Compound 7 [103]	Compound 8 [103]
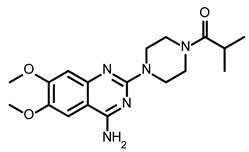	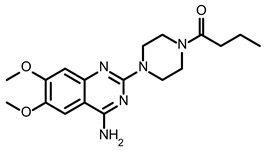
Compound 10 [103]